# Bilayer Heterostructure Electrolytes Were Prepared by a UV-Curing Process for High Temperature Lithium-Ion Batteries

**DOI:** 10.3390/polym16212972

**Published:** 2024-10-23

**Authors:** Qiankun Hun, Lingxiao Lan, Xuanan Lu, Qicheng Hu, Xinghua Liang, Yifeng Guo, Yujiang Wang

**Affiliations:** 1 Guangxi Key Laboratory of Automobile Components and Vehicle Technology, Guangxi University of Science & Technology, Liuzhou 545006, China; 13383371921@163.com (Q.H.); llx2685062@163.com (L.L.); a15007751851@163.com (X.L.); lxh304@126.com (X.L.); wangyujiang@gxust.edu.cn (Y.W.); 2 School of Mechanical Engineering, Chengdu University, Chengdu 610106, China

**Keywords:** bilayer heterostructure electrolyte, high-temperature performance, UV curing process, lithium-ion battery

## Abstract

Solid-state electrolytes are widely anticipated to revitalize high-energy-density and high-safety lithium-ion batteries. However, low ionic conductivity and high interfacial resistance at room temperature pose challenges for their practical application. In this work, the dual-matrix concept is applied to the design of a bilayer heterogeneous structure. The electrolyte in contact with the cathode blends PVDF-HFP and oxidation-resistant PAN. In contrast, the electrolyte in contact with the anode blends PVDF-HFP and reduction-resistant PEO. A UV-curing process was used to fabricate the bilayer heterostructure electrolyte. The heterostructure electrolyte exhibits an ionic conductivity of 4.27 × 10^−4^ S/cm and a Li^+^ transference number of 0.68 at room temperature. Additionally, when assembled into LiFePO_4_/CPEs/Li batteries, it shows a high initial discharge capacity at room temperature (168 mAh g^−1^ at 0.1 C and 60 mAh g^−1^ at 2 C), with a capacity retention of 93.3% after 100 cycles at a current density of 0.2 C. Notably, at 60 °C, the battery maintains a discharge capacity of 90 mAh g^−1^ at 2 C, with a capacity retention of 97.4% after 100 cycles at 0.2 C. Therefore, solid-state batteries using this bilayer heterogeneous structure electrolyte demonstrate promising performance, including effective capacity output and cycling stability.

## 1. Introduction

The invention of lithium-ion batteries (LIBs) has significantly improved our quality of life through the widespread use of portable electronic devices and electric vehicles [1,2]. LIBs are among the most promising energy storage devices due to their high energy density, long cycle life, and low self-discharge rates [3,4]. However, the inclusion of liquid electrolytes has compromised their safety and limited their operational conditions, constraining their development. The interface chemistry and electrochemical instability of organic-liquid electrolytes lead to the formation of lithium dendrites on the lithium metal surface [5]. These dendrites can puncture the separator, causing short circuits and thermal runaway, potentially resulting in fires and accidents [6]. These issues can be addressed by using solid-state electrolytes (SSEs). Although the development of solid-state electrolytes is promising, with excellent electrochemical stability, good environmental adaptability, and mechanical stability, the limitations at the electrode interface have yet to be thoroughly studied [7].

Solid-state electrolytes (SSEs) are mainly classified into three types: inorganic solid electrolytes (ISEs), solid polymer electrolytes (SPEs), and composite polymer electrolytes (CPEs). ISEs exhibit high ionic conductivity and can suppress lithium dendrites; however, their poor electrochemical stability and interface contact issues limit their application. The inherent rigidity and brittleness of ISEs also hinder their widespread use [8,9,10]. Pioneering and subsequent research efforts have focused on reducing the intrinsic interfacial (grain boundary) resistance [11] of ISEs and improving the interface design between ISEs and electrodes [12]. SPEs show good interface contact and ease of processing [13]. Unfortunately, at room temperature, SPEs have low ionic conductivity and poor electrochemical stability, which similarly limits their practical applications. As battery electrolytes, it is challenging for SPEs to meet the requirements of LIBs, particularly with regard to acceptable ionic conductivity [14]. The electrochemical stability and mechanical properties of solid-state electrolytes and their compatibility with lithium metal and cathode materials present a challenge. Developing new high-performance solid polymer electrolytes often involves a trade-off between improving ionic conductivity and maintaining mechanical strength.

Among SSEs, composite polymer electrolytes (CPEs) combine the advantages of ISEs and SPEs and are considered the most promising candidates for LIBs. Specifically, CPEs based on matrices such as polymethyl methacrylate (PMMA) [15,16], poly(ethylene oxide) (PEO) [17,18], polyacrylonitrile (PAN) [19,20], and poly(vinylidene fluoride)-hexafluoropropylene (PVDF-HFP) [21,22] have been extensively studied. In practice, the choice of electrolyte materials is crucial. Low-dimensional inorganic fillers [23], oxide fillers [24,25], functional fillers [26,27], and structural designs [28] are widely applied to enhance the performance of CPEs. Lithium lanthanum zirconium oxide (LLZTO), known for its rapid lithium-ion conduction, offers excellent mechanical properties and effective ion transport pathways. Blending [29], comb-like formation [30], and polymer alloying [31] can suppress polymer chain crystallization, which helps increase conductivity. Polymer blending can enhance mechanical strength and is essential for developing new polymer materials. It is also a practical technique for designing materials with various properties [32]. Specifically, by altering the composition of blends, polymer blending can lead to a range of excellent properties in the final CPEs products. Careful selection of host polymers can provide the additional benefit of reduced crystallinity. Combinations of proton-conducting and proton-receptive polymers can form macromolecular complexes in aqueous or organic media. Additionally, adding salts can affect polymer chain arrangement and conductivity. To achieve high lithium-ion conductivity in CPEs: (i) the polymer should be compatible with inorganic salts; (ii) the polymer should provide interconnected polar domains as conduction pathways; and (iii) the polymer should have suitable interactions with the carrier ions to prevent their complete trapping. Lithium perchlorate (LiClO_4_), with its low lattice energy (723 kJ mol^−1^) and good thermal stability, helps increase charge carriers. Compared to other lithium salts, LiClO_4_ has a larger anion size, making it the chosen ionic salt for this study.

However, gaps between CPEs and electrodes have not been fundamentally resolved. Therefore, the Goodenough group proposed a bilayer polymer electrolyte to match high-voltage cathodes [33]. Wang et al. used LAGP-PEO to prepare a composite solid electrolyte that could inhibit the growth of lithium dendrites. They studied the effect of different PEO contents on the performance of the anode [34]. Duan et al. developed a multilayer composite solid-state electrolyte that inhibits the formation of lithium dendrites at a current density of 2 mA cm^−1^ with a polarization voltage of less than 40 mV for lithium batteries within 1000 h [28]. Mu et al. designed a bilayer solid-state electrolyte composed of PAN and LLTO nanofibers in contact with the positive electrode and PEO and LLZTO in contact with the negative electrode, which has good electrochemical properties [35]. Shalu et al. discussed the recent advances of ionic liquid-based polymer electrolytes (ILPEs) to create innovative polymer electrolytes (PEs) and their applications in energy generation and storage [36]. Abhimanyu et al. discussed the different types of anodes and their properties in lithium-ion batteries (LIBs) and sodium-ion batteries (SIBs) [37]. Ma et al. summarized the high-voltage cathode materials and their matching solid-state electrolytes and analyzed the interface problem from a new perspective (corrosion) [38].

In this study, CPEs were designed and synthesized using a UV-curing process, and the effects of different polymer blending ratios on CPEs were investigated. For contact with the cathode, PVDF-HFP was blended with antioxidant PAN, while for contact with the anode, PVDF-HFP was blended with reductant-resistant PEO. Experiments showed that a blending ratio of 3:7 for both PVDF-HFP with PAN and PVDF-HFP with PEO achieved the highest ionic conductivity. This technology holds significant promise for application in next-generation high-performance laboratories.

## 2. Materials and Methods

### 2.1. Materials

PVDF, PVDF-HFP (Mn = 600,000, Arkema, Colombes, France), poly(acrylonitrile) (PAN, Mw = 85,000, Shanghai, China), poly(ethylene oxide) (PEO, Mv = 600,000, Sigma Aldrich, Shanghai, China), lithium perchlorate (LiClO_4_) (99.99%, Aladdin Chemical Co., Shanghai, China), N-methyl pyrrolidone (NMP, ≥99.9%), Super-P (≥99.5%), and LiFePO_4_ (LFP, ≥99.5%) were all from Krud in Shanghai, China. Plasticizers included trimethylolpropane ethoxylate triacrylate (ETPTA, Mn = 428, Macklin, Shanghai, China), polyurethane acrylate (PUA, RYOJI, Frankfurt, Germany), and 2-hydroxy-2-methylpropiophenone (1173, Cambridge, Beijing, China).

### 2.2. Preparation of CPEs

Preparation of PAP and PEP: According to the ratios of PVDF-HFP and PAN (or PEO) of 1:9, 3:7, 5:5, 7:3, and 9:1, a certain amount of polymer was weighed, dissolved in DMF on a magnetic stirrer at 45 °C (or 55 °C), LiClO_4_ was added, and LLZTO (10% compared to polymer) was added after dissolving, and stirred for six hours. Add a certain amount of ETPTA and PUA, stir for 5 min, add 1173, use a 100 μm film scraper to create a solid electrolyte film, vacuum dry for one hour, and cut into 19 mm discs for later use. The proportions of solid-state electrolyte membranes were defined as PAP 1:9, PAP 3:7, PAP 5:5, PAP 7:3, and PAP 9:1 (PEP 1:9, PEP 3:7, PEP 5:5, PEP 7:3, and PEP 9:1).

Preparation of PAP/PEP: After the preparation of the PAP solid electrolyte membrane is completed, PEP is prepared with a film scraper and then cut into 19 mm for later use after vacuum drying, which is defined as PAP/PEP.

### 2.3. Material Characterization

The cut CPEs were analyzed using an X-ray diffractometer (XRD, DX-2700, Dandong, China, Cu-Kα, 40 kV × 30 mA). Surface and cross-section analyses of the CPEs were performed using a scanning electron microscope (SEM, Phenom spectra G2, Shanghai, China), and the cross-section of the CPEs was obtained by liquid nitrogen embrittlement, and the CPEs were gold-sprayed prior to testing. The tensile strength of CPEs cut into rectangles was tested using a universal testing machine (WDW-5, Tenson, Jinan, China). The cut CPEs were subjected to thermogravimetric analysis (TGA), which was carried out under a nitrogen atmosphere using a TG analysis system (NetzschF3Tarsus, Beyern, Germany) with a temperature range of 30~800 °C and a heating rate of 10 °C min^−1^. The functional groups on the cropped CPE’s surfaces were determined using Fourier transform infrared spectroscopy (FTIR, spectrum 100, PerkinElmer, Waltham, MA, USA) in the range of 500~4000 cm^−1^.

### 2.4. Electrochemical Properties

Electrochemical impedance spectroscopy (EIS) is a measurement of different temperatures at an open-circuit voltage (OCV) of 0.1 Hz~1 MHz with an amplitude of 10 mV, sandwiching CPEs between two smooth SS electrodes. The ionic conductivity (*σ*) of the different electrolytes was calculated according to the EIS results and Equation (1):

(1) $$\sigma =\frac{L}{RS}$$

where *R* is the bulk resistance obtained by EIS, and *L* and *S* are the thickness and effective area of the CPEs, respectively. The activation energy (*E_a_*) of each solid electrolyte is calculated using the Arrhenius formula (Equation (2)):

(2) $$\sigma (T)=A\exp\left[-\frac{E_{a}}{RT}\right]$$

where *A* is the exponential prefactor, *E_a_* is the activation energy of the activated ion skipping conduction process, and *T* is the absolute temperature. The electrochemical windows of various CPEs were tested using linear scanning voltammetry (LSV) over a voltage range of 5 mV^−1^. SS/CPEs/Li are assembled in CR2025 coin batteries for LSV testing. Two polished lithium foils were used in a symmetrical cell to study the stability of various solid-state electrolytes against the lithium anode. When measuring the *t_Li_^+^* (lithium-ion transference number), the same symmetrical cells assembled with different electrolytes were also measured using the chronoampering method, with a polarization of 10 mV applied to the cells for 4000 s. At the same time, the AC impedance spectrum before and after polarization was recorded at a 10 mV oscillation voltage with a frequency of 0.1 Hz~1 MHz, and *t_Li_^+^* was calculated using Equation (3):

(3) $$t_{{Li}^{+}}=\frac{I_{s}\left(\Delta V-R_{0}I_{0}\right)}{I_{0}\left(\Delta V-R_{s}I_{s}\right)}$$

where *I*_0_ and *I_S_* represent the initial and steady-state currents, respectively; *R*_0_ and *R_S_* represent the charge transfer resistance of the electrolyte system before and after polarization; and Δ*V* is the oscillation voltage of 10 mV. LFP/CPEs/Li are assembled in CR2025 coin batteries for cyclic voltammetry (CV) testing. The scan rate is 0.2 mV s^−1^.

Rate and cycle tests of the batteries were carried out on battery test systems (Neware, Dongguan, China). Commercial LFP is the positive active material, and the lithium sheet is the negative electrode. LFP, PVDF, and conductive carbon have a weight-to-mass ratio of 8:1:1. Place it in the solvent, apply the obtained solution on the aluminum foil, vacuum dry it for 36 h, and cut it into a 14 mm disc for later use. The test was carried out in a 2.8 to 4.0 V voltage range.

## 3. Results and Discussion

**Figure 1:**
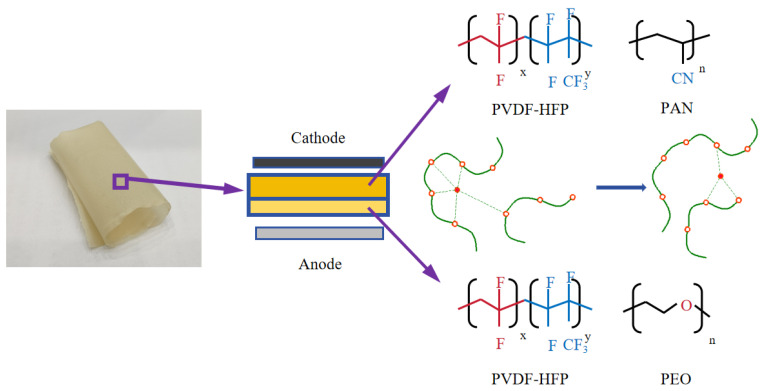
Schematic diagram of the structure of PAP/PEP membranes and schematic diagram of ion transport.

Figure 1 illustrates the structure of the PAP/PEP membrane and its ionic transport. Lithium-ion migration in the electrolyte primarily occurs under an electric field, interacting with various electronegative groups. To illustrate the expected electrochemical performance of PAP/PEP, Figure 1 presents a schematic diagram of ion transport. In PAP/PEP, lithium ions first enter the PAP 3:7 membrane, where oxygen vacancies in LLZTO facilitate ion transport in conjunction with polar groups on the polymer chains, distributing along the high-binding energy interface between LLZTO and the matrix. At the interface, strong hydrogen bonds enable carriers to rapidly and stably pass through into the PEP 3:7 membrane, which contains reduction-resistant PEO, facilitating uniform lithium-ion deposition on the electrode through the combined action of polymer chains and LLZTO. The intermediate polymer can withstand volume changes and undergo chemical reactions to form a solid electrolyte interphase (SEI). This layered design is expected to enhance the electrochemical performance of the electrolyte and guarantee a sound interface with metallic Li. The flexibility of the PAP/PEP membrane ensures excellent processing characteristics for solid-state electrolytes. PAN is a hydrophilic polymer with nitrile (–CN) functional groups linked to the polyethylene chain. The lone pair of electrons on the nitrogen atom can act as a hydrogen bond acceptor, and a significant dipole moment is induced between the electron-rich nitrogen and electron-poor carbon atoms [39]. This inhibits bond rotation and consequently stiffens the polymer chain. PEO plays a crucial role in developing lithium-ion batteries, primarily due to the transient cross-linking between cations and ether oxygens, which facilitates ion dissociation. However, PEO tends to crystallize at room temperature, which limits ionic transport. PVDF−HFP, due to the introduction of the amorphous HFP phase, exhibits lower crystallinity and higher free volume, creating ion channels that enhance ionic conductivity. In summary, this bilayer heterogeneous structure improves electrochemical stability while ensuring effective ion transport.

**Figure 2:**
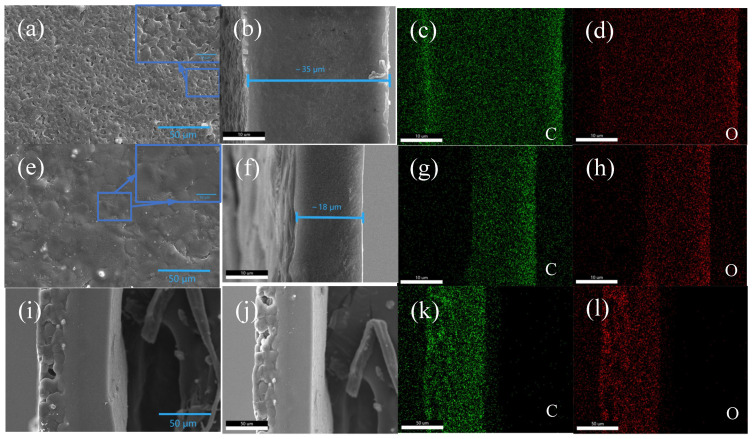
(**a**,**b**) The SEM surface and cross-section of PAP 3:7 membranes. (**c**,**d**) The mapping of C and O, respectively, distributed in the PAP 3:7 membranes. (**e**,**f**) The SEM surface and cross-section of PEP 3:7 membranes. (**g**,**h**) The mapping of C, and O, respectively, distributed in the PAP 3:7 membranes. (**i**,**j**) The SEM cross-section of PAP/PEP membranes. (**k**,**l**) The mapping of C, and O, respectively, distributed in the PAP/PEP membranes.

Scanning electron microscopy (SEM) was used to analyze the morphology of the electrolyte. The surface of the PAP 3:7 membrane, shown in Figure 2a, displays a uniform porous structure with an approximate thickness of 35 μm (Figure 2b). Additionally, the EDS results in Figure 2c,d indicate a uniform distribution of carbon (C) and oxygen (O). As shown in Figure 2e, the surface of the PEP 3:7 membrane is smoother and more uniform, with a thickness of approximately 18 μm (Figure 2f). Notably, there are no apparent gaps at the interface of the PEP 3:7 membrane, indicating good adhesion and uniform contact, which helps to ensure a consistent Li^+^ flux at the internal solid electrolyte interface. Corresponding EDS spectra (Figure 2g,h) show a uniform distribution of C and O. Figure 2i,j present cross-sectional images of the PAP/PEP membrane, demonstrating the tight contact between the two layers. In contrast, the EDS results in Figure 2k,l reveal a uniform distribution of C and O.

**Figure 3:**
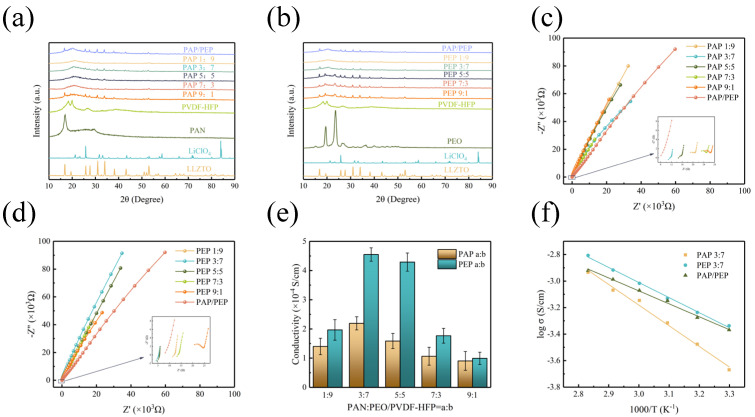
(**a**,**b**) XRD patterns of CPEs with various components. (**c**,**d**) Nyquist plot of SS/CPEs/SS battery tests at room temperature (inset: enlarged impedance spectra). (**e**) Ionic conductivity values of CPEs at different temperatures. (**f**) Arrhenius plot for PAP 3:7, PEP 3:7, and PAP/PEP as a function of the different temperatures.

Typical X−ray diffraction (XRD) patterns of the components and solid-state electrolyte membranes are shown in Figure 3a,b. PVDF-HFP exhibits two distinct peaks near 18° and 20° in 2θ, PAN shows a prominent peak at 16° in 2θ, and PEO has noticeable peaks at 19° and 23° in 2θ. The addition of lithium salts causes significant disruption in the polymer structure, resulting in a decreased crystallinity of the polymers. Amorphous polymers, with their flexible leading chains, exhibit higher ionic diffusivity and conductivity due to their amorphous nature. The peaks corresponding to the lithium salts nearly disappear in the polymer complexes. This indicates good compatibility between the polymer and lithium salts, suggesting adequate mixing at the molecular level and favorable interactions between the polymer and salt functional groups.

The optimal ratios of PVDF-HFP with PAN or PEO, tested at 1:9, 3:7, 5:5, 7:3, and 9:1, were initially determined based on the ionic conductivity of the solid electrolytes. Nyquist plots for the various ratios of solid electrolytes are shown in Figure 3c,d. For PAP membranes at ratios of 1:9, 3:7, 5:5, 7:3, and 9:1, the volume resistances were 20 Ω, 11 Ω, 15 Ω, 25 Ω, and 26 Ω, respectively. For PEP membranes, the volume resistances were 12 Ω, 5 Ω, 6 Ω, 13 Ω, and 24 Ω, respectively. Ionic conductivities of the solid electrolytes at various ratios are shown in Figure 3e. The highest ionic conductivities for both PAP and PEP membranes were observed at the 3:7 ratio, with values of 2.14 × 10^−4^ S/cm and 4.6 × 10^−4^ S/cm, respectively. Increasing the amount of PAN or PEO further decreases the ionic conductivity of the electrolyte, likely due to local aggregation of the polymer when PAN or PEO is excessive. In summary, PAP 3:7 and PEP 3:7 were selected as solid electrolytes to form a bilayer solid electrolyte, PAP/PEP, with a room-temperature ionic conductivity of 4.27 × 10^−4^ S/cm. Table 1 shows a comparison of PAP/PEP membranes with existing solid-state electrolytes. These three solid electrolytes were used for subsequent tests. Activation energies calculated using the Arrhenius equation were 0.33 eV, 0.31 eV, and 0.29 eV for PAP 3:7, PEP 3:7, and PAP/PEP, respectively, indicating that PAP/PEP has lower lithium-ion migration energy consumption (Figure 3f).

**Figure 4:**
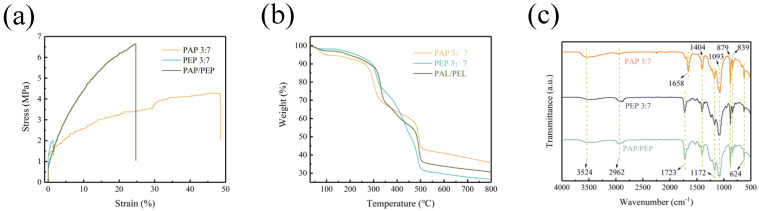
(**a**) Stress−strain curves of PAP 3:7, PEP 3:7, and PAP/PEP membranes. (**b**) TGA curves of the PAP 3:7, PEP 3:7, and PAP/PEP membranes. (**c**) FTIR spectra of the PAP 3:7, PEP 3:7, and PAP/PEP membranes.

The mechanical stability of lithium−ion batteries (LIBs) largely depends on the strength of the solid electrolyte. Mechanical failures occur when internal stresses exceed the strength of the solid electrolyte, affecting the battery’s electrochemical performance. As shown in Figure 4a, the tensile strength of the PAP 3:7 membrane is 4.27 MPa with an elongation of 48.6%; the tensile strength of the PEP 3:7 membrane is 2 MPa with an elongation of 1.6%, while the PAP/PEP membrane exhibits a tensile strength of 6.65 MPa and an elongation of 24.7%. Although the elongation is lower than that of the PAP 3:7 membrane, the bilayer structure of the PAP/PEP membrane provides the highest tensile strength, which is crucial for suppressing lithium dendrite growth during cycling. We also conducted thermogravimetric analysis (TGA) to assess the thermal stability of the solid electrolyte membranes (Figure 4b). Below 300 °C, the decomposition is attributed to the residual solvents and plasticizers, such as ETPTA, in the solid electrolyte membranes. Notably, between 300 °C and 500 °C, all three membranes exhibit similar decomposition due to the breakdown of polymers in the solid electrolyte membranes. As the temperature increases, at 800 °C, the PEP 3:7 membrane has the lowest residue (26.7%), while the PAP 3:7 membrane has the highest residue (35.7%). The PAP/PEP membrane, composed of PAP 3:7 and PEP 3:7, shows intermediate residue (30.9%), indicating that this promising solid electrolyte remains viable even at high temperatures.

FTIR spectra were used to investigate the interactions between the polymer matrix, LiClO_4_, and LLZTO (Figure 4c). The broad peak at 3524 cm^−1^ confirms the coordination between Li⁺ and ether oxygens through the C–O–C linkage. Peaks at 2962 cm^−1^ and 1723 cm^−1^ correspond to C–H stretching and C=O stretching in the PEO matrix. The peak at 1658 cm^−1^ corresponds to C=O stretching in PAN. Peaks at 1404 cm^−1^ (C–H bending in CH_2_), 1172 cm^−1^ (CF_3_ symmetric stretching), 1093 cm^−1^ (C–C stretching), 879 cm^−1^ (C–C skeletal vibration), 839 cm^−1^ (C–H bending in CH_2_), and 624 cm^−1^ correspond to the presence of ClO_4_^−^ anions in the complexes, indicating coordination between the polymer and lithium salt. The FTIR results suggest that the bilayer structure design enhances interactions between the –C–H groups and lithium ions, with the Lewis acidic –C–H groups providing channels for lithium ion transport.

**Figure 5:**
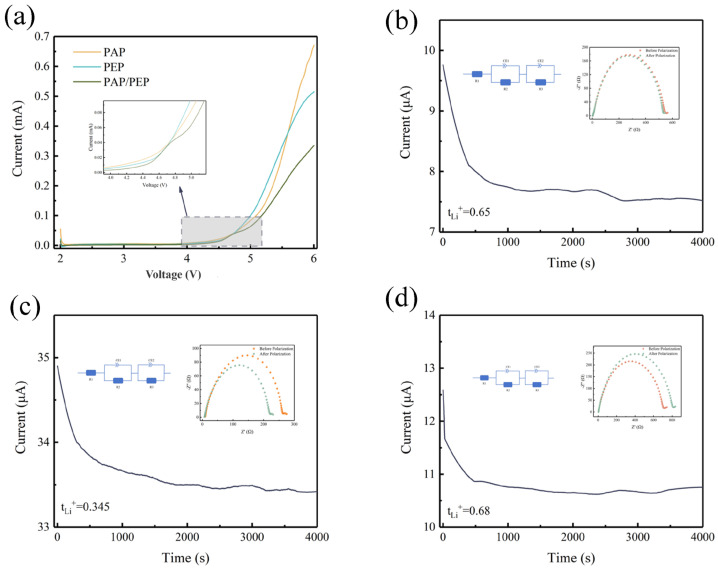
(**a**) LSV profiles of CPEs membranes. (**b**–**d**) DC polarization curves of the Li/PAP 3:7/Li Li/PEP 3:7/Li and Li/PAP/PEP/Li batteries at a polarization voltage of 10 mV.

Beyond Li^+^ conductivity, the electrochemical stability and Li^+^ migration rate of solid-state electrolytes are crucial for the feasibility of solid-state batteries. A wide voltage window for solid-state electrolytes (SSEs) is essential in practical applications. This study used linear sweep voltammetry (LSV) to determine the electrochemical window of Li/CPEs/SS. The experimental results, shown in Figure 5a, indicate that PAP/PEP has the most comprehensive voltage range, starting to decompose around 4.5 V, which is higher than PAP 3:7 at 4 V and PEP 3:7 at 4.1 V. This improvement is attributed to the design of the bilayer structure. A wide operating voltage range ensures the capability to meet the demands of high-energy-density lithium-ion batteries (LIBs) with high-voltage cathodes. The Li^+^ transference number (*t_Li_^+^*) is another critical parameter for evaluating ion conductivity effectiveness. A lower *t_Li_^+^* means a more significant proportion of anion migration and total ion conductivity, leading to increased concentration polarization and higher resistance detrimental to LIBs. The *t_Li_^+^* values for PAP 3:7 and PEP 3:7 solid-state electrolytes are 0.65 and 0.345, respectively (Figure 5b,c). In contrast, the bilayer structure of PAP/PEP results in a higher *t_Li_^+^* of 0.68 (Figure 5d), indicating that PAP/PEP solid-state electrolytes may reduce polarization and enhance the rapid charge-discharge capabilities of the battery system. Table 2 lists the *t_Li_^+^* values for PAP 3:7 films, PEP 3:7 films, and PAP/PEP films.

**Figure 6:**
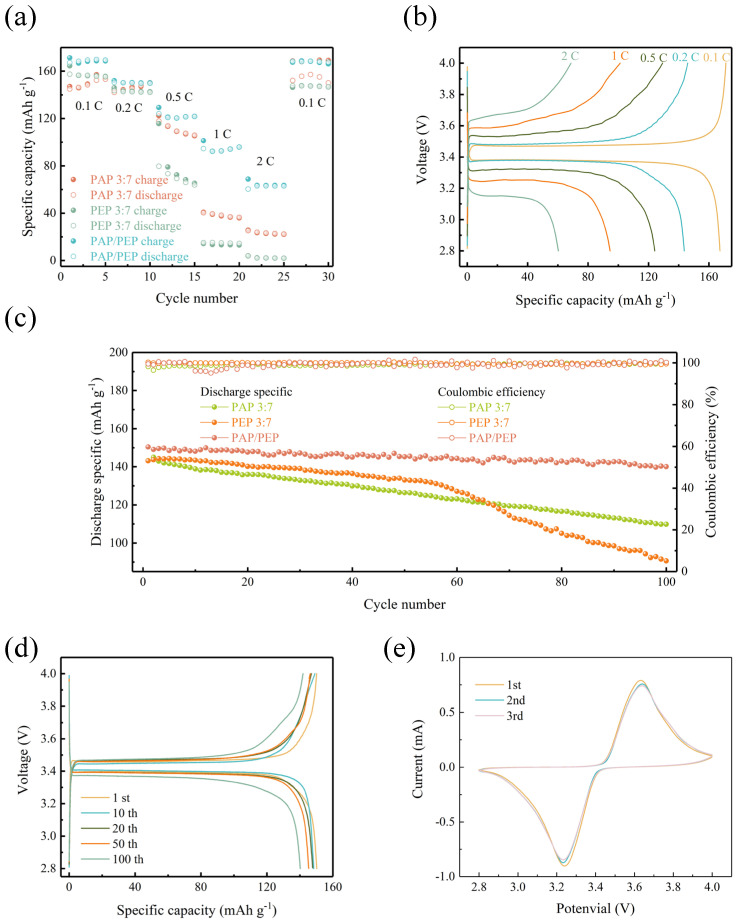
(**a**) The rate capability of PAP 3:7−based LFP/Li, PEP 3:7−based LFP/Li, and PAP/PEP−based LFP/Li batteries at 25 °C. (**b**) Galvanostatic charge−discharge curves of PAP/PEP−based LFP/Li batteries at 0.1, 0.2, 0.5, 1 C, and 2 C. (**c**) Cycling performances of PAP 3:7−based LFP/Li, PEP 3:7−based LFP/Li, and PAP/PEP−based LFP/Li batteries at 25 °C. (**d**) Galvanostatic charge−discharge curves of PAP/PEP−based LFP/Li batteries at 0.2 C,100 cycles. (**e**) CV curves of lithium−ion batteries with PAP/PEP electrolyte at a scan rate of 0.2 mV s^−1^.

To evaluate the practical feasibility of various solid−state electrolytes, 2025−coin cells were assembled with LiFePO_4_ as the cathode and Li metal as the anode and tested at room temperature (25 °C). The rate performance of batteries with three different electrolytes was investigated at current densities ranging from 0.1 C to 2 C, as shown in Figure 6a. Thanks to the unique structure of the PAP/PEP electrolyte, where the oxidation−resistant PAN contacts the cathode and the reduction−resistant PEO contacts the anode, batteries with the PAP/PEP electrolyte achieve the highest rate capability. The battery delivers reversible capacities of 168, 150, 124, 94, and 60 mAh g^−1^ at 0.1 C, 0.2 C, 0.5 C, 1 C, and 2 C, respectively, in the first cycle. Notably, when the current density is suddenly reduced back to 0.1 C, it maintains a high discharge capacity of 168 mAh g^−1^. In contrast, due to significant polarization in the PAP 3:7 and PEP 3:7 electrolytes, the LFP delivers lower discharge capacities at corresponding current densities, specifically 145, 142, 117, 40, and 25 mAh g^−1^ for PAP 3:7 and 157, 143, 79, 15, and 3 mAh g^−1^ for PEP 3:7. When returned to 0.1 C, the PAP 3:7 electrolyte recovers to its initial discharge capacity but remains lower than that of PAP/PEP. Similarly, the PEP 3:7 electrolyte can only recover 93.6% of its initial discharge capacity. The charge−discharge curves for PAP/PEP at corresponding current densities are shown in Figure 6b. The observed low overpotentials indicate minimal polarization, which is favorable for long−term cycling. As the current intensity increases, the polarization voltage rises slowly. Additionally, the room−temperature cycling performance of batteries with the three electrolytes was tested at a loading of 2.4 mg cm^−2^ (Figure 6c). At a current density of 0.2 C, the PAP/PEP−based battery retains a reversible capacity of 140 mAh g^−1^ after 100 cycles, with a high−capacity retention rate of 93.3% and nearly 100% stable Coulombic efficiency. The charge−discharge curves for the 1st, 10th, 20th, 50th, and 100th cycles of the PAP/PEP battery are shown in Figure 6d. Unfortunately, the batteries based on PAP 3:7 and PEP 3:7 start with a lower initial capacity of 143 mAh g^−1^, showing capacity retention rates of 76.2% and 62.9%, respectively. Thanks to the interfacial reactions’ chemical/electrochemical balance, batteries using the PAP/PEP electrolyte exhibit outstanding cycling performance. Table 3 shows the performance of PAP/PEP membranes compared to existing solid−state electrolytes. The cyclic voltammetry (CV) curve of a PAP/PEP−based cell (Figure 6e) shows an oxidation peak of 3.65 V and a reduction peak of 3.25 V at a scan rate of 0.2 mV s^−1^. The overlap was good during the three cycles, indicating that LiFePO_4_ had excellent reversibility and high cycle stability during the redox reaction.

**Figure 7:**
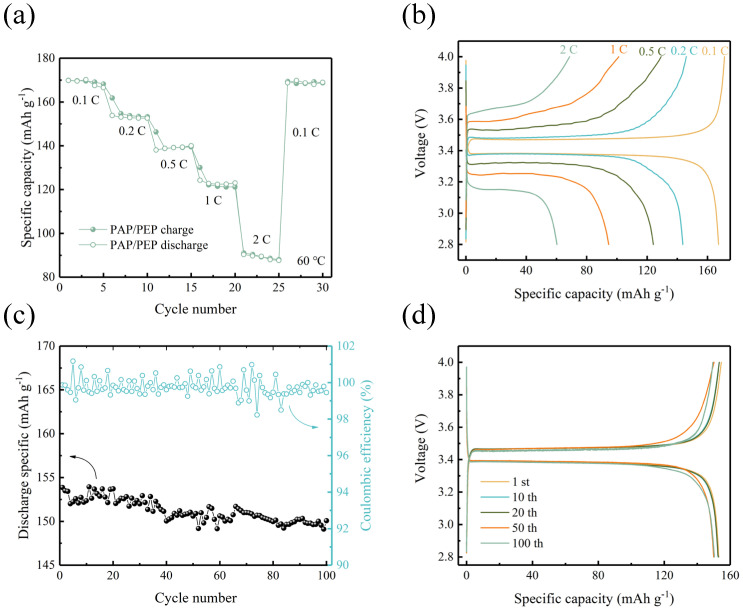
(**a**) The rate capability of PAP/PEP−based LFP/Li batteries at 25 °C. (**b**) Galvanostatic charge−discharge curves of PAP/PEP−based LFP/Li batteries at 0.1, 0.2, 0.5, 1 C, and 2 C. (**c**) Cycling performances of PAP/PEP−based batteries at 0.2 C, 60 °C. (**d**) Galvanostatic charge−discharge curves of PAP/PEP−based LFP/Li batteries at 0.2 C, 100 cycles.

To meet the performance requirements for operation over a wide temperature range, we conducted rate performance tests on PAP/PEP batteries at 60 °C (Figure 7a). The higher temperature accelerates kinetics, resulting in an initial discharge capacity of 169 mAh g^−1^ at a current density of 0.1 C, with a polarization voltage of 100 mV (Figure 7b). Notably, at a current density of 2 C, the discharge capacity is higher at 60 °C compared to 25 °C (90 vs. 60 mAh g^−1^). Overall, the PAP/PEP battery demonstrates excellent rate capability at 60 °C. Additionally, we performed cycling tests at 60 °C (Figure 7c). At a current density of 0.2 C, the initial discharge capacity is 154 mAh g^−1^, with a reasonable capacity retention rate of 97.4% after 100 cycles. The polarization remains almost unchanged after cycling, indicating high cycling stability.

## 4. Conclusions

In summary, we have designed and successfully fabricated a bilayer heterogeneous electrolyte composed of a PVDF-HFP and an oxidation-resistant PAN blend in contact with the cathode and a PVDF-HFP and a reduction-resistant PEO blend in contact with the anode. This design enhances lithium-ion batteries’ safety, functionality, and electrochemical performance. At room temperature, the solid-state electrolyte exhibits an ionic conductivity of 4.27 × 10^−4^ S/cm, a Li^+^ transference number of 0.68, and an electrochemical window of 4.5 V, with a tensile strength of 6.65 MPa. Furthermore, the solid-state electrolyte enables LiFePO_4_/CPEs/Li batteries to achieve excellent performance, including high capacity (168 mAh g^−1^ at 0.1 C and 60 mAh g^−1^ at 2 C) and good cycling stability (93.3% capacity retention and 99.2% Coulombic efficiency after 100 cycles at 0.2 C). Notably, at 60 °C, the battery maintains a discharge capacity of 90 mAh g^−1^ at 2 C, with 97.4% capacity retention and 99.7% Coulombic efficiency after 100 cycles. Therefore, solid-state electrolytes based on this design concept have the potential to exhibit promising electrochemical performance and advance the practical development of safe, high-energy lithium-ion batteries.

## Figures and Tables

**Table 1:** Comparison with existing solid-state electrolytes.

SSEs	Ionic Conductivity (×10^−4^ S/cm)	Ref.
PLP	2.57 (30 °C)	[40]
PPPL-10	4 (25 °C)	[41]
SSCEs	2.91 (25 °C)	[42]
PAP/PEP	4.27 (RT)	This work

**Table 2:** Experimentally measured parameters of solid electrolytes and their lithium-ion transfer numbers (*t_Li_^+^*) calculated at room temperature.

CPEs	*I*_0_ (μA)	*I_s_* (μA)	*R*_0_ (Ω)	*R_s_* (Ω)	*t_Li_* ^+^
PAP 3:7	9.76	7.5	508.8	535.8	0.65
PEP 3:7	34.9	33.4	257.3	215.7	0.345
PAP/PEP	12.58	10.75	712.8	810.7	0.68

**Table 3:** Performance comparison with existing solid-state electrolytes.

SSEs	0.1 C (mAh g^−1^)	0.2 C (mAh g^−1^)	0.5 C (mAh g^−1^)	After Cycles (mAh g^−1^)	Ref.
PLP	120	152.5	103	127.4 (0.2 C, 100)	[40]
GPFIL3	/	145	131	117 (0.5 C, 350)	[43]
GPE−8	96	93	87.5	83.2 (0.5 C, 300)	[44]
PAP/PEP	168	150	124	140 (0.2 C, 100)	This work

## Data Availability

Data are contained within the article.

## References

[1] Savisha M., Abreeza M., Kam S.L., Dita F., Rinaldi M.R., Sylvia A.P., Ramisha R., Chin H.C., Nurfanizan A., Agung N. (2024). Review of bioresource-based conductive composites for portable flexible electronic devices. Renew. Sustain. Energy Rev..

[2] Wei L., Xu X., Xi K., Lei Y., Cheng X., Shi X.B., Wu H.H., Gao Y.F. (2024). Ultralong Cycling and Interfacial Regulation of Bilayer Heterogeneous Composite Solid-State Electrolytes in Lithium Metal Batteries. ACS Appl. Mater. Interfaces.

[3] Wu D., Luo M., Yang R., Hu X., Lu C. (2023). Achieve High Dielectric and Energy-Storage Density Properties by Employing Cyanoethyl Cellulose as Fillers in PVDF-Based Polymer Composites. Materials.

[4] Kim H., Lee S.H., Kim J.M., Yoon C.S., Sun Y.K. (2023). High-Energy-Density, Long-Life Li-Metal Batteries via Application of External Pressure. ACS Energy Lett..

[5] Ko C., Chen C., Chen C., Chen K. (2023). Influence of inhomogeneity of lithium-ion transport within the anode/electrolyte interface on mossy lithium formation. J. Power Sources.

[6] Manthiram A., Yu X., Wang S. (2017). Lithium battery chemistries enabled by solid-state electrolytes. Nat. Rev. Mater..

[7] Liang J.N., Luo J., Sun Q., Yang X.F., Li R.Y., Sun X.L. (2019). Recent progress on solid-state hybrid electrolytes for solid-state lithium batteries. Energy Storage Mater..

[8] Daems K., Yadav P., Dermenci K.B., Mierlo J.V., Berecibar M. (2024). Advances in inorganic, polymer and composite electrolytes: Mechanisms of Lithium-ion transport and pathways to enhanced performance. Renew. Sustain. Energy Rev..

[9] Sun J.C., Liu C.B., Liu H.Y., Li J.W., Zheng P.L., Zheng Y., Liu Z.H. (2023). Advances in Ordered Architecture Design of Composite Solid Electrolytes for Solid-State Lithium Batteries. Chem. Rec..

[10] Kim J.S., Yoon G., Kim S., Sugata S., Yashiro N., Suzuki S., Lee M.J., Kim R., Badding M., Song Z. (2023). Surface engineering of inorganic solid-state electrolytes via interlayers strategy for developing long-cycling quasi-all-solid-state lithium batteries. Nat. Commun..

[11] Li Y.T., Chen X., Dolocan A., Cui Z.M., Xin S., Xue L.G., Xu H.H., Park K., Goodenough J. (2018). Garnet Electrolyte with an Ultralow Interfacial Resistance for Li-Metal Batteries. J. Am. Chem. Soc..

[12] Liang J.Y., Zeng X.X., Zhang X.D., Zuo T.T., Yan M., Yin Y.X., Shi J.L., Wu X.W., Guo Y.G., Wan L.J. (2019). Engineering Janus Interfaces of Ceramic Electrolyte via Distinct Functional Polymers for Stable High-Voltage Li-Metal Batteries. J. Am. Chem. Soc..

[13] Choudhury S., Stalin S., Vu D., Warren A., Deng Y., Biswal P., Archer L. (2019). Solid-state polymer electrolytes for high-performance lithium metal batteries. Nat. Commun..

[14] Grewal M., Ishibashi K., Hara M., Ishizaki Y., Nagano S., Yabu H. (2023). Effect of the Poly(ethylene glycol) Diacrylate (PEGDA) Molecular Weight on Ionic Conductivities in Solvent-Free Photo-Cross-Linked Solid Polymer Electrolytes. Langmuir.

[15] Salam O.A., Hamad H.A., Eltokhy M.A.R., Ali A.I., Son J.Y., Ramzy G. (2024). A comparative study of PMMA/PEG polymer nanocomposites doped with different oxides nanoparticles for potential optoelectronic applications. Sci. Rep..

[16] Huang J., Li Z.Y., Liu F.X., Wei W., Xu X.F., Liu Z.J. (2023). Preparation of micron-sized silicon monoxide (SiOx)@carbon nanotube (CNT)/polymethyl methacrylate (PMMA) composite capsules as Li-ion battery anodes via a novel pickering emulsion template method. Powder Technol..

[17] Yang L., Zhang H., Xia E.J., Wu Y.M., Li Z.C. (2023). PEO/Li2ZrO3 composite electrolyte for solid-state rechargeable lithium battery. J. Energy Storage.

[18] Liu Y.Y., Han L.F., Liao C., Yu H., Kan Y.C., Hu Y. (2023). Ultra-thin, non-combustible PEO polymer solid electrolyte for high safety polymer lithium metal batteries. Chem. Eng. J..

[19] Liang Y., Yang Q. (2022). Zeolitic imidazolate framework-8 derivative/carbonized polyacrylonitrile composite filled in Ni foam as a self-supporting anode for lithium-ion batteries. Ionics.

[20] Fang X., Liu C.Y., Peng H., Li Y., Yang Y.G. (2024). Gel electrolyte and anode material derived from electro-spun polyacrylonitrile/polysilsesquioxane composite and application in advanced lithium-ion batteries. Mater. Lett..

[21] Zhao Q., Wu X., Li S., Zheng Q., Jiang S., Xu Y., He B., Ma L., Luo Y., Wang Y. (2023). Boosting Thermal and Mechanical Properties: Achieving High-Safety Separator Chemically Bonded with Nano TiN Particles for High Performance Lithium-Ion Batteries. Small.

[22] Waqas M., Soomro A.M., Ali S., Ashraf H., Chan A.S., Kumar S., Choi K.H. (2021). A highly efficient composite separator embedded with colloidal lanthanum oxide nanocrystals for high-temperature lithium-ion batteries. Int. J. Energy Res..

[23] Sun J., Yao X., Li Y., Zhang Q., Hou C., Shi Q., Wang H. (2020). Facilitating Interfacial Stability Via Bilayer Heterostructure Solid Electrolyte Toward High-energy, Safe and Adaptable Lithium Batteries. Adv. Energy Mater..

[24] Xiong X.P., Wang Y.G. (2024). A gel polymer electrolyte membrane of polyhedral oligomeric silsesquioxane cross-linked poly(vinylidene fluoride-hexafluoropropylene) for lithium-ion battery. Chem. Eng. J..

[25] Xu G.J., Zhao M., Xie B., Wang X., Jiang M.M., Guan P., Han P.X., Cui G.L. (2021). A rigid-flexible coupling gel polymer electrolyte towards high safety flexible Li-Ion battery. J. Power Sources.

[26] Liu C.C., Wu B.R., Liu T., Zhang Y.X., Cui J.W., Huang L.J., Tan G.Q., Zhang L., Su Y.F., Wu F. (2024). Metal-organic frameworks and their composites for advanced lithium-ion batteries: Synthesis, progress and prospects. J. Energy Chem..

[27] Ren G.Y., Cai F.S., Wang S.C., Luo Z.Q., Yuan Z.H. (2023). Iodine doping induced activation of covalent organic framework cathodes for Li-ion batteries. RSC Adv..

[28] Duan H., Fan M., Chen W.P., Li J.Y., Wang P.F., Wang W.P., Shi J.L., Yin Y.X., Wan L.J., Guo Y.G. (2019). Extended Electrochemical Window of Solid Electrolytes via Heterogeneous Multilayered Structure for High-Voltage Lithium Metal Batteries. Adv. Mater..

[29] Zhao M.K., Zuo X.X., Ma X.D., Xiao X., Liu J.S., Nan J.M. (2017). Self-supported PVdF/P(VC-VAc) blended polymer electrolytes for LiNi0.5Mn1.5O4/Li batteries. J. Membr. Sci..

[30] Fan Y., Wang H., Chen S., Hou Y., Wang S. (2023). An In Situ Prepared Comb-like Polycaprolactone-Based Gel Electrolyte for High-Performance Lithium Metal Batteries. Materials.

[31] Miao W.W., Wang J.X., Li G.X., Liu S.L., Luo X.G. (2023). Superior thermal stability of PVA/cellulose composite membranes for lithium-ion battery separators prepared by impregnation method with noncovalent cross-linking of intermolecular multiple hydrogen-bonds. J. Energy Storage.

[32] Chen Z., Guan M.D., Cheng Y.W., Li H., Ji G.J., Chen H., Fu X.G., Awuye D.E., Zhu Y.B., Yin X.C. (2023). Boehmite-enhanced poly(vinylidene fluoride-co-hexafluoropropylene)/polyacrylonitrile (PVDF-HFP/PAN) coaxial electrospun nanofiber hybrid membrane: A superior separator for lithium-ion batteries. Adv. Compos. Hybrid. Mater..

[33] Zhou W.D., Wang S.F., Li Y.T., Xin S., Manthiram A., Goodenough J.B. (2016). Plating a dendrite-free lithium anode with a polymer/ceramic/polymer sandwich electrolyte. JACS.

[34] Wang C.H., Yang Y.F., Liu X.J., Zhong H., Xu H., Xu Z.B., Shao H.X., Ding F. (2017). Suppression of Lithium Dendrite Formation by Using LAGPPEO (LiTFSI) Composite Solid Electrolyte and Lithium Metal Anode Modified by PEO (LiTFSI) in All-Solid-State Lithium Batteries. ACS Appl. Mater. Interfaces.

[35] Mu S., Huang W.L., Sun W.H., Zhao N., Jia M.Y., Bi Z.J., Guo X.X. (2021). Heterogeneous electrolyte membranes enabling doubleside stable interfaces for solid lithium batteries. J. Energy Chem..

[36] Shalu, Singh R.K., Dhar R. (2021). Momentous past and key advancements in ionic liquid mediated polymer electrolyte for application in energy storage. Int. J. Energy Res..

[37] Abhimanyu K.P., Ashish B. (2023). A review on anode materials for lithium/sodium-ion batteries. J. Energy Chem..

[38] Ma M.M., Zhang M.H., Jiang B.T., Du Y., Hu B.C., Sun C.G. (2023). A review of all-solid-state electrolytes for lithium batteries: High-voltage cathode materials, solid-state electrolytes and electrode–electrolyte interfaces. Mater. Chem. Front..

[39] Vahid V., Mehmet E.P., Borte K.M., Ismail K. (2023). Polyacrylonitrile in the preparation of separation membranes: A review. Ind. Eng. Chem. Res..

[40] Gao L.X., Tang B., Jiang H.Y., Xie Z.J., Wei J.P., Zhou Z. (2022). Fiber-Reinforced Composite Polymer Electrolytes for Solid-State Lithium Batteries. Adv. Sustain. Syst.

[41] Wang Y., Chen Z., Wu Y.X., Li Y., Yue Z.Y., Chen M.H. (2023). PVDF-HFP/PAN/PDA@LLZTO Composite Solid Electrolyte Enabling Reinforced Safety and Outstanding Low-Temperature Performance for Quasi-Solid-State Lithium Metal Batteries. ACS Appl. Mater. Interfaces.

[42] Zou K., Cai Z., Ke X., Wang K.L., Tan X.Q., Luo D.D., Huang F., Wang C.Y., Cheng J.K., Xiao R.G. (2023). Electrochemical properties of LATP ceramic electrolyte doped with LiBiO_3_ sintering additive and its derived sandwich structure composite solid electrolyte. Ionics.

[43] Cui Y., Yu G.F., Liu R.L., Miao D.G., Wu D.C. (2023). Quasi-Solid-State Composite Electrolytes with Multifunctional 2D Molecular Brush Fillers for Long-Cycling Lithium Metal Batteries. Chin. J. Chem..

[44] Wang X.T., Wang X.X., Chen J.J., Zhao Y., Mao Z.Y., Wang D.J. (2021). Durable sodium battery composed of conductive Ti_3_C_2_T_x_ MXene modified gel polymer electrolyte. Solid State Ion..

